# Supplementary material for: Rotational Spectrum and Conformational Analysis of Perillartine: Insights into the Structure–Sweetness Relationship
Source: Molecules. 2022 Mar 16;27(6):1924. doi: 10.3390/molecules27061924 (PMC8954681; doi:10.3390/molecules27061924)
Supplement: Supplementary file 1 [file molecules-27-01924-s001.zip › molecules-1609858-supplementary.pdf]

Supporting information for:

Rotational Spectrum and Conformational Analysis of Perillartine: Insights into the Structure- Sweetness Relationship

G. Juárez[1], M. Sanz-Novo[1], J. L. Alonso[1], E. R. Alonso[1], and I. León[1,*]

[1]Grupo de Espectroscopía Molecular (GEM), Edificio Quifima, Laboratorios de Espectroscopia y Bioespectroscopia, Unidad Asociada CSIC, Parque Científico UVa, Universidad de Valladolid, 47011 Valladolid, Spain.

*Corresponding Author:

Iker León Ona, ileon@uva.es

phones: +34 983 186344 / +34 983 186349

web: http://www.gem.uva.es/

Figure S1. Different views of the predicted lowest-energy conformers of perillartine below 2000 cm^{-1} relative to the global minimum.

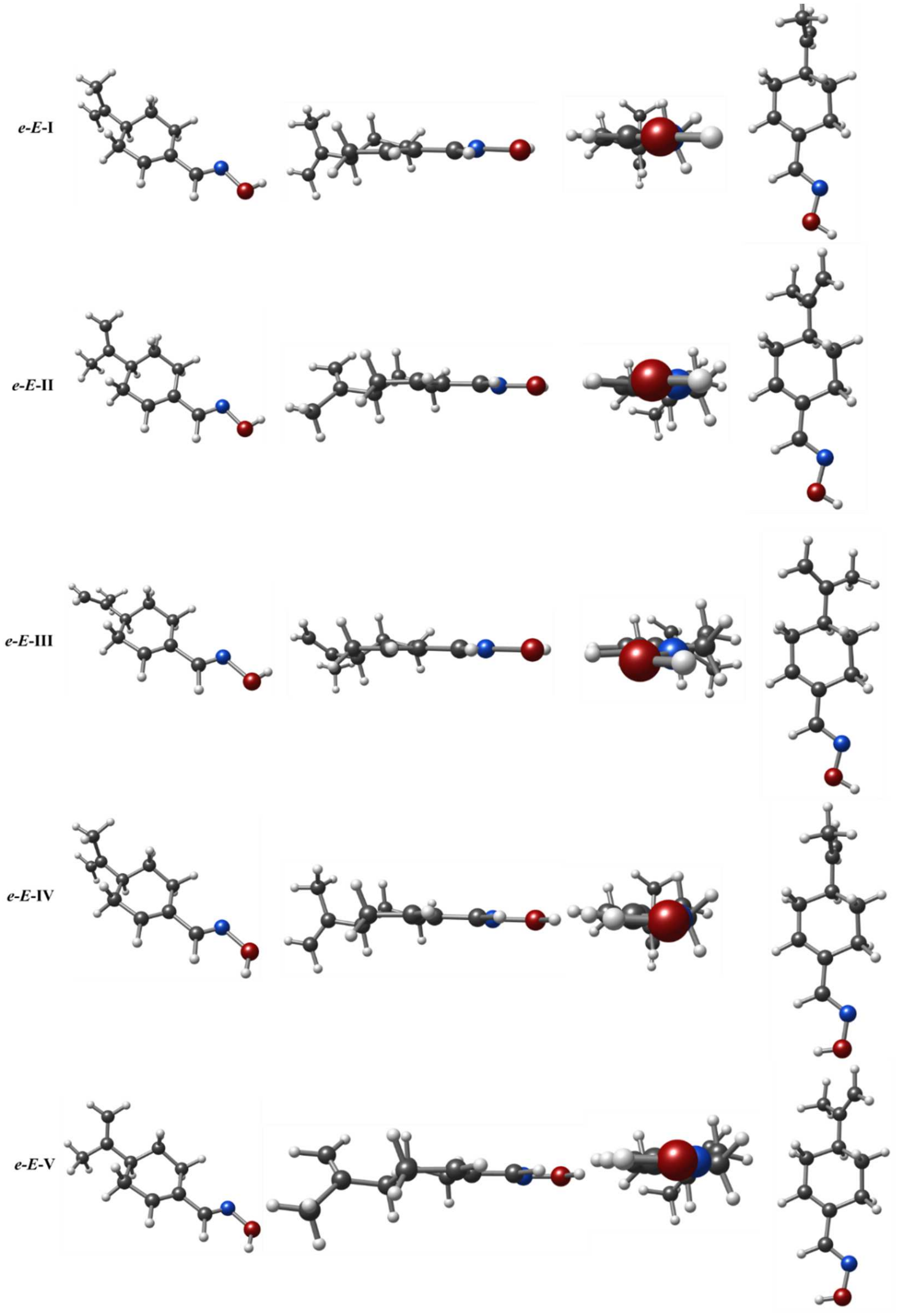

Figure S1. Continuation.

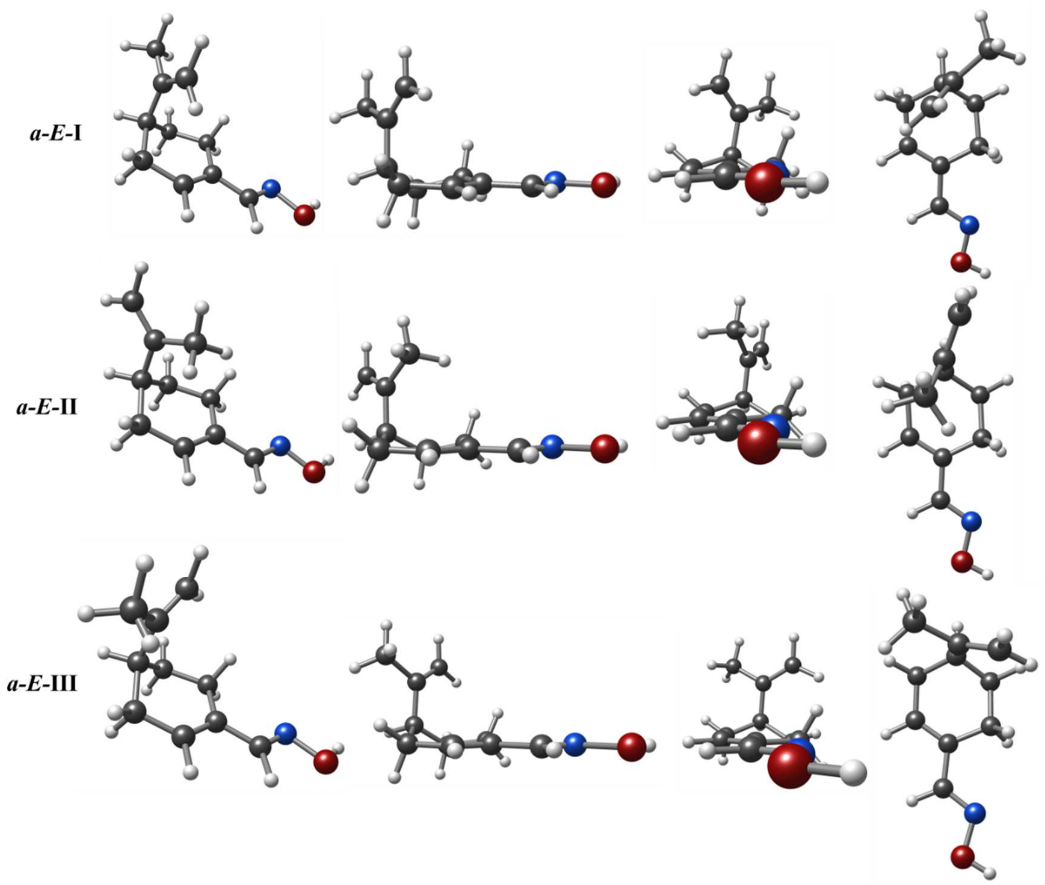

Figure S1. Continuation.

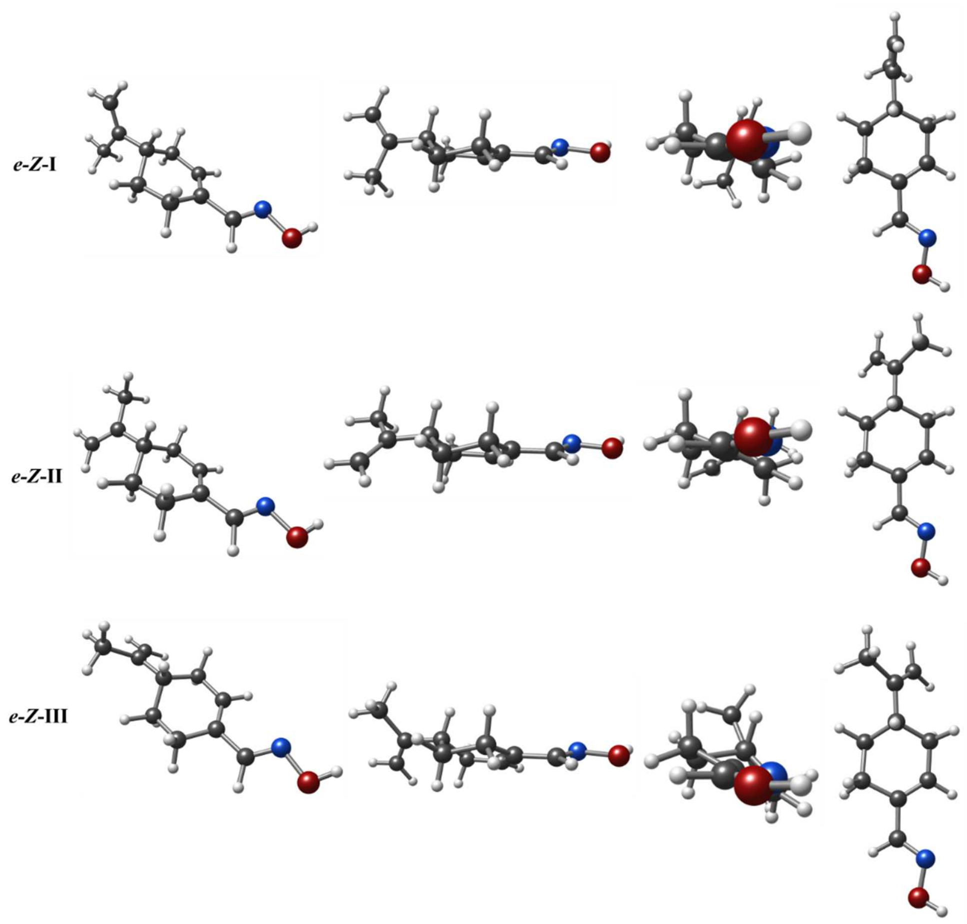

Figure S1. Continuation.

***a*-*Z*-I**

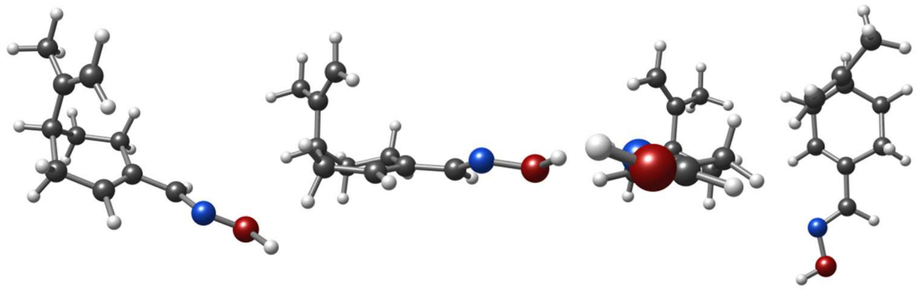

Figure S2. Potential Energy Scan (PES) rotating the methyl group, i.e. the C-C-C-H dihedral angle, of structure *e*-*E*-I. The barrier height is ~550 cm^{-1} and, therefore, no splitting is expected due to internal rotation.

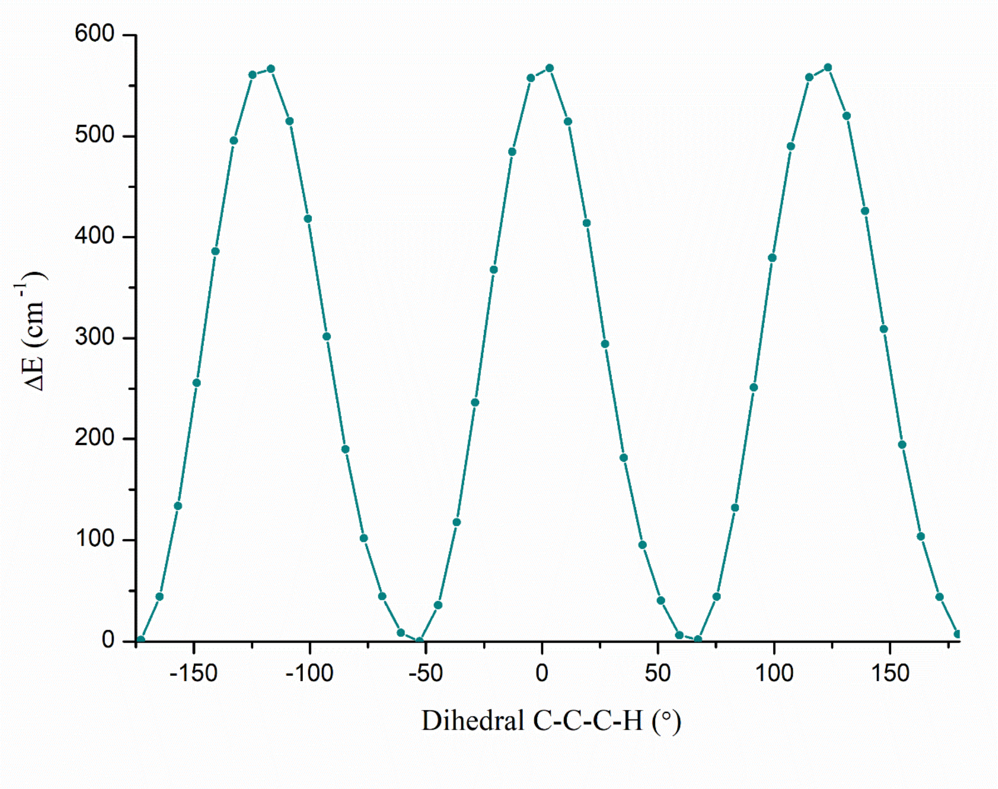

Table S1. Measured frequencies and residuals (in MHz) for the transitions of rotamer I of perillartine.

J'	K'_{-1}	K'_{+1}	*J''*	K''_{-1}	K''_{+1}	ν_{obs}	$\nu_{obs} - \nu_{cal}$
4	0	4	3	0	3	2943.525	0.019
5	1	5	4	1	4	3646.145	-0.004
5	0	5	4	0	4	3678.973	-0.006
5	1	4	4	1	3	3713.514	0.022
6	1	6	5	1	5	4375.226	-0.008
6	0	6	5	0	5	4414.146	-0.037
6	3	3	5	3	2	4416.320	-0.048
6	1	5	5	1	4	4456.089	0.049
7	1	7	6	1	6	5104.256	0.018
7	0	7	6	0	6	5149.059	-0.006
7	2	6	6	2	5	5151.683	0.023
7	2	5	6	2	4	5154.663	-0.010
7	1	6	6	1	5	5198.561	0.057
8	1	8	7	1	7	5833.192	0.038
8	0	8	7	0	7	5883.568	-0.004
8	2	7	7	2	6	5887.386	-0.045
8	2	6	7	2	5	5891.953	0.004
8	1	7	7	1	6	5940.871	0.000
9	1	9	8	1	8	6561.983	0.017
9	0	9	8	0	8	6617.625	-0.029
9	2	8	8	2	7	6623.117	-0.014
9	2	7	8	2	6	6629.548	-0.031
9	1	8	8	1	7	6683.123	-0.001
10	1	10	9	1	9	7290.697	0.032
10	0	10	9	0	9	7351.251	-0.008
10	2	9	9	2	8	7358.735	-0.016
10	1	9	9	1	8	7425.254	0.005

Table S2. Measured frequencies and residuals (in MHz) for the transitions of rotamer II of perillartine.

J'	*K'*$_{-1}$	*K'*$_{+1}$	*J''*	*K''*$_{-1}$	*K''*$_{+1}$	ν_{obs}	$\nu_{obs} - \nu_{cal}$
4	0	4	3	0	3	2920.931	0.036
5	1	5	4	1	4	3583.791	-0.014
5	0	5	4	0	4	3649.433	0.003
5	1	4	4	1	3	3722.105	0.014
2	1	2	1	0	1	3966.178	0.007
6	1	6	5	1	5	4299.960	-0.003
6	0	6	5	0	5	4376.815	-0.033
6	2	4	5	2	3	4391.526	-0.053
3	1	3	2	0	2	4655.549	0.000
7	1	7	6	1	6	5015.805	0.001
7	0	7	6	0	6	5102.907	-0.028
7	2	6	6	2	5	5113.751	-0.004
7	2	5	6	2	4	5126.338	0.010
7	1	6	6	1	5	5209.311	0.017
4	1	4	3	0	3	5331.472	0.006
8	1	8	7	1	7	5731.280	-0.001
8	0	8	7	0	7	5827.494	0.008
8	2	7	7	2	6	5843.498	-0.043
8	2	6	7	2	5	5862.347	-0.001
8	1	7	7	1	6	5952.314	0.024
5	1	5	4	0	4	5994.362	-0.014
9	1	9	8	1	8	6446.368	0.013
9	0	9	8	0	8	6550.346	0.029
9	2	8	8	2	7	6573.023	-0.002
9	3	7	8	3	6	6580.599	0.018
9	3	6	8	3	5	6581.110	-0.001
9	1	8	8	1	7	6694.795	0.010
10	1	10	9	1	9	7160.998	0.009
10	0	10	9	0	9	7271.299	0.033
10	2	8	9	2	7	7338.716	-0.053
10	1	9	9	1	8	7436.706	0.004

Table S3. Measured frequencies and residuals (in MHz) for the transitions of rotamer III of perillartine.

J'	*K'*$_{-1}$	*K'*$_{+1}$	*J'*	*K"*$_{-1}$	*K"*$_{+1}$	ν_{obs}	$\nu_{obs} - \nu_{cal}$
3	1	3	2	1	2	2839.691	-0.056
4	0	4	3	0	3	3821.708	0.032
5	0	5	4	0	4	4775.607	0.000
5	1	4	4	1	3	4825.170	0.013
6	1	6	5	1	5	5678.206	0.021
6	0	6	5	0	5	5728.539	-0.018
6	2	5	5	2	4	5734.581	-0.019
7	1	7	6	1	6	6623.841	0.006
7	0	7	6	0	6	6680.353	0.010
7	2	6	6	2	5	6689.888	0.024
7	2	5	6	2	4	6700.932	0.001
7	1	6	6	1	5	6753.772	0.010
8	1	8	7	1	7	7569.163	-0.010
8	1	7	7	1	6	7717.491	-0.026

Table S4. Measured frequencies and residuals (in MHz) for the transitions of rotamer IV of perillartine.

J'	***K'***$_{-1}$	***K'***$_{+1}$	***J'***	***K"***$_{-1}$	***K"***$_{+1}$	ν_{obs}	$\nu_{obs} - \nu_{cal}$
4	0	4	3	0	3	2936.217	-0.017
5	1	5	4	1	4	3590.951	-0.008
5	0	5	4	0	4	3667.831	-0.004
5	1	4	4	1	3	3754.997	0.035
6	1	6	5	1	5	4308.292	0.014
6	0	6	5	0	5	4397.759	-0.052
6	1	5	5	1	4	4505.066	0.047
7	1	7	6	1	6	5025.151	0.012
7	1	6	6	1	5	5254.529	-0.017
8	1	8	7	1	7	5741.488	0.006
8	0	8	7	0	7	5851.713	0.010
8	1	7	7	1	6	6003.428	-0.012
9	1	9	8	1	8	6457.264	0.012

Table S5. Cartesian coordinates for the *e*-*E*-I structure of perillartine optimized at B3LYP-GD3BJ/6-311++G(d,p).

Center Number	Atomic Number	X	Y	Z
1	6	-0.741038000	-1.139629000	-0.288848000
2	6	0.739099000	-1.082479000	0.096013000
3	6	1.307977000	0.310268000	-0.034608000
4	6	0.520516000	1.393974000	-0.148862000
5	6	-0.978911000	1.352594000	-0.130155000
6	6	-1.525514000	0.003448000	0.376514000
7	1	-1.166104000	-2.105090000	-0.000257000
8	1	-0.834065000	-1.060278000	-1.377507000
9	1	1.325357000	-1.768032000	-0.520896000
10	1	0.879443000	-1.425867000	1.128228000
11	1	0.984601000	2.371433000	-0.257488000
12	1	-1.363397000	2.163735000	0.497542000
13	1	-1.354188000	1.560543000	-1.141130000
14	1	-1.321598000	-0.043003000	1.452449000
15	6	2.754222000	0.485055000	-0.028780000
16	1	3.161693000	1.494433000	-0.112447000
17	7	3.538832000	-0.518294000	0.078152000
18	8	4.883545000	-0.107824000	0.067176000
19	1	5.364085000	-0.938179000	0.150419000
20	6	-3.024787000	-0.108150000	0.194614000
21	6	-3.844376000	-0.076379000	1.247449000
22	1	-4.920918000	-0.146157000	1.133929000
23	1	-3.462962000	0.019628000	2.258551000
24	6	-3.552613000	-0.259600000	-1.209144000
25	1	-4.643549000	-0.254632000	-1.220750000
26	1	-3.202860000	0.545977000	-1.862430000
27	1	-3.213163000	-1.198470000	-1.657841000

Table S6. Cartesian coordinates for the *e*-*E*-II structure of perillartine optimized at B3LYP-GD3BJ/6-311++G(d,p).

Center Number	Atomic Number	X	Y	Z
1	6	-0.690007000	-1.248476000	-0.121686000
2	6	0.775853000	-1.069314000	0.280740000
3	6	1.315125000	0.283361000	-0.116354000
4	6	0.502128000	1.295770000	-0.463090000
5	6	-0.995471000	1.213798000	-0.461187000
6	6	-1.526550000	-0.031432000	0.282991000
7	1	-1.091595000	-2.154127000	0.339537000
8	1	-0.752277000	-1.386316000	-1.207048000
9	1	1.396225000	-1.847845000	-0.169916000
10	1	0.893785000	-1.193380000	1.364225000
11	1	0.941182000	2.244305000	-0.763279000
12	1	-1.401537000	2.128136000	-0.016977000
13	1	-1.363916000	1.198412000	-1.496745000
14	1	-1.354401000	0.142296000	1.355407000
15	6	2.755716000	0.499134000	-0.119206000
16	1	3.139532000	1.481450000	-0.401388000
17	7	3.562486000	-0.436875000	0.207525000
18	8	4.896148000	0.002519000	0.138041000
19	1	5.395317000	-0.778633000	0.398773000
20	6	-3.023383000	-0.179284000	0.093559000
21	6	-3.580207000	-1.179020000	-0.592732000
22	1	-4.656469000	-1.244269000	-0.709690000
23	1	-2.995046000	-1.961761000	-1.058351000
24	6	-3.868012000	0.894105000	0.732885000
25	1	-4.932595000	0.685258000	0.616336000
26	1	-3.647760000	0.975820000	1.803056000
27	1	-3.668189000	1.876778000	0.293530000

Table S7. Cartesian coordinates for the *a*-*E*-I structure of perillartine optimized at B3LYP-GD3BJ/6-311++G(d,p).

**Center Number**	**Atomic Number**	X	Y	Z
1	6	0.947718000	-0.205364000	-1.559083000
2	6	-0.352182000	-0.662235000	-0.894867000
3	6	-1.016192000	0.457919000	-0.133537000
4	6	-0.343537000	1.565712000	0.223116000
5	6	1.106231000	1.808099000	-0.080769000
6	6	1.855039000	0.555463000	-0.564850000
7	1	0.713773000	0.466505000	-2.391922000
8	1	1.472890000	-1.062703000	-1.985900000
9	1	-0.156745000	-1.488346000	-0.201288000
10	1	-1.047738000	-1.055356000	-1.639798000
11	1	-0.869991000	2.350730000	0.761069000
12	1	1.174604000	2.584378000	-0.855038000
13	1	1.603446000	2.230547000	0.798435000
14	1	2.743943000	0.887438000	-1.114794000
15	6	-2.416252000	0.328031000	0.244784000
16	1	-2.885239000	1.138316000	0.806523000
17	7	-3.090034000	-0.713542000	-0.063649000
18	8	-4.410278000	-0.616104000	0.411680000
19	1	-4.805701000	-1.445224000	0.122800000
20	6	2.348743000	-0.361551000	0.542072000
21	6	1.919078000	-0.303284000	1.803488000
22	1	2.299986000	-0.986747000	2.554513000
23	1	1.172136000	0.408503000	2.130948000
24	6	3.377042000	-1.379045000	0.115971000
25	1	4.249183000	-0.887093000	-0.329035000
26	1	3.715909000	-1.980514000	0.960796000
27	1	2.977875000	-2.059286000	-0.643155000

Table S8. Cartesian coordinates for the *e*-*E*-III structure of perillartine optimized at B3LYP-GD3BJ/6-311++G(d,p).

Center Number	Atomic Number	X	Y	Z
1	6	0.759041000	-0.914302000	0.688939000
2	6	-0.717981000	-1.014209000	0.299141000
3	6	-1.320838000	0.333619000	-0.015270000
4	6	-0.561125000	1.425988000	-0.207582000
5	6	0.938564000	1.430078000	-0.152550000
6	6	1.526726000	0.019468000	-0.269261000
7	1	1.202732000	-1.912289000	0.700150000
8	1	0.853231000	-0.514231000	1.704624000
9	1	-1.295650000	-1.487964000	1.097038000
10	1	-0.839199000	-1.666291000	-0.574536000
11	1	-1.049385000	2.375453000	-0.413405000
12	1	1.338701000	2.072697000	-0.943902000
13	1	1.250808000	1.893806000	0.792136000
14	1	1.320700000	-0.333734000	-1.289894000
15	6	-2.768825000	0.450638000	-0.122244000
16	1	-3.201214000	1.424464000	-0.360064000
17	7	-3.526102000	-0.564289000	0.051719000
18	8	-4.878828000	-0.214606000	-0.107120000
19	1	-5.337226000	-1.046849000	0.049571000
20	6	3.026564000	-0.069820000	-0.067868000
21	6	3.751362000	0.856388000	0.562735000
22	1	4.820911000	0.733270000	0.693161000
23	1	3.319428000	1.762290000	0.968557000
24	6	3.671859000	-1.307113000	-0.639299000
25	1	4.741421000	-1.337344000	-0.425906000
26	1	3.219083000	-2.218706000	-0.236846000
27	1	3.536917000	-1.342114000	-1.726202000

Additional Information: B3LYP vs MP2

The main difference between the four main conformers is that structures *e-E*-I, *e-E*-II, and *a-E*-III have the allyl group in an equatorial position, while structure *a-E*-I has the allyl group in an axial position. It is worth noting that calculations using the MP2 level also predicts the same four structures as the most stable structures, but with structure *a-E*-I, the third most stable structure with B3LYP, as the global minimum (see Table S9). This fact is interesting as it lets us to benchmark current methodologies. As described in the main manuscript, using selected transitions of the experimental results we can estimate the relative populations of the conformers, and set structure 1 as the global minimum, with structure 3 being slightly less populated. Therefore, B3LYP-GD3 gives a better description of the energetics. Finally, we would also like to highlight the importance of adding Grimme Dispersions with Becke-Johnson damping as the calculations without including them estimate structure *a-E*-I being too high in energy, at 560 cm^{-1}, which would result in a low population of this conformer to allow its detection. The conformational panorama of this molecule is another illustrative case of the robustness of rotational spectroscopy, not only for a definitive structural characterization, but also to contrast, benchmark, and validate the results provided by high-level computational chemistry.

Table S9. Theoretical spectroscopic parameters for low-energy conformers of perillartine computed at the MP2/6-311++G(d,p) level of theory.

Parameters	*a-E*-I	*e-E*-I	*e-E*-II	*e-E*-III	*a-E*-II	*a-E*-III	*a-Z*-I	*e-Z*-I	*e-Z*-II	*e-Z*-III	*e-E*-IV	*e-E*-V
A[1]	1750	2878	2908	2794	1852	1636	1809	2806	2743	2768	2870	2900
B	497	376	380	383	490	508	491	375	384	378	376	380
C	476	362	352	354	475	497	478	361	352	357	362	352
$\|\mu_a\|$[2]	1.1	0.8	0.9	0.9	0.7	1.1	1.2	0.8	0.8	0.8	1.7	1.8
$\|\mu_b\|$	0.1	0.1	0.2	0.3	0.4	0.4	0.4	0.2	0.2	0.4	2.7	2.9
$\|\mu_c\|$	0.6	0.4	0.2	0.1	0.0	0.3	0.1	0.2	0.2	0.2	0.7	0.7
ΔE[3]	0	99	270	359	538	713	1138	1307	1459	1578	1916	2089
ΔE_{ZPE}[4]	0	40	208	295	583	790	1114	1223	1374	1489	1751	1922
ΔG[5]	37	0	135	223	676	859	1102	1149	1273	1377	1718	1856

[1] *A*, *B*, and *C* represent the rotational constants (in MHz); [2] $|\mu_a|$, $|\mu_b|$ and $|\mu_c|$ are the absolute values of the electric dipole moment components (in D). [3] Relative energies (in cm^{-1}) concerning the global minimum; [4] Relative energies (in cm^{-1}) with respect to the global minimum, taking into account the zero-point energy (ZPE); [5] Gibbs energies (in cm^{-1}) calculated at 298 K.
